# Inflammatory caspase substrate specificities

**DOI:** 10.1128/mbio.02975-23

**Published:** 2024-06-05

**Authors:** Patrick M. Exconde, Christopher M. Bourne, Madhura Kulkarni, Bohdana M. Discher, Cornelius Y. Taabazuing

**Affiliations:** 1 Department of Biochemistry and Biophysics, University of Pennsylvania Perelman School of Medicine, Philadelphia, Pennsylvania, USA; The Ohio State University, Columbus, Ohio, USA

**Keywords:** inflammasomes, inflammatory caspases, caspase-1, caspase-4, caspase-5, caspase-11, IL-1β, IL-18, pyroptosis, cytokines, caspase substrates

## Abstract

Caspases are a family of cysteine proteases that act as molecular scissors to cleave substrates and regulate biological processes such as programmed cell death and inflammation. Extensive efforts have been made to identify caspase substrates and to determine factors that dictate substrate specificity. Thousands of putative substrates have been identified for caspases that regulate an immunologically silent type of cell death known as apoptosis, but less is known about substrates of the inflammatory caspases that regulate an immunostimulatory type of cell death called pyroptosis. Furthermore, much of our understanding of caspase substrate specificities is derived from work done with peptide substrates, which do not often translate to native protein substrates. Our knowledge of inflammatory caspase biology and substrates has recently expanded and here, we discuss the recent advances in our understanding of caspase substrate specificities, with a focus on inflammatory caspases. We highlight new substrates that have been discovered and discuss the factors that engender specificity. Recent evidence suggests that inflammatory caspases likely utilize two binding interfaces to recognize and process substrates, the active site and a conserved exosite.

## INTRODUCTION

Caspases (cysteine-dependent aspartic proteases) are a family of structurally related proteases that are best known for their role in executing programmed cell death pathways and regulating inflammation (1–4). A subset of caspases (caspase-2, -3, -6, -7, -8, -9, and -10) are known to predominantly execute an immunologically silent type of programmed cell death known as apoptosis, and another subset (caspase-1, -4, -5, and -11) are known to mediate an immunostimulatory type of cell death called pyroptosis. It is estimated that the substrate repertoire of caspases numbers in the thousands, with some caspases being more well-characterized than others (4–8). This review will focus on the most recent advances in our understanding of the substrates cleaved by inflammatory caspases, caspase-1, -4, -5, and -11 (CASP1/4/5/11), and the factors that engender specificity for their substrates. We also briefly discuss the crosstalk that exists between apoptotic and inflammatory caspases.

Caspases utilize cysteine (C) residues as a nucleophile to catalyze the cleavage of peptide bonds and cleave after aspartic acid (D) residues (2, 5, 9). Cleavage has also been reported following glutamate (E) and phosphorylated serine residues for some caspases, but processing is reduced ~3-fold compared to cleavage after D residues and virtually nothing is known about the identity or function of native substrates cleaved after phosphoserine residues (5). Interestingly, replacing D with E in caspase peptide substrates can result in a 400- to 20,000-fold decrease in processing (9). In contrast, cleavage activity drops by only ~3-fold in protein substrates, suggesting that there may be additional factors involved in tuning the substrate specificities in protein substrates that can help overcome the stringent requirement for cleaving after D residues.

### Identification of the first inflammatory caspase

In 1992, two independent groups identified the protease that cleaves interleukin-1β, and named it interleukin converting enzyme (ICE) (10, 11). Later, ICE was renamed caspase-1 (CASP1) as more knowledge of the enzyme family was revealed (1). At the time, it was understood that CASP1 was involved in cell death and played a critical role in inflammation, but it was not until 2015 that the key substrate of CASP1 and the effector protein that mediates cell death, gasdermin D (GSDMD), was discovered (12–14). Importantly, it was revealed that GSDMD is a substrate of all the inflammatory caspases and activation of GSDMD to induce cell death occurs following caspase activation on large macromolecular signaling complexes known as inflammasomes.

### Canonical inflammasomes

**Fig 1:**
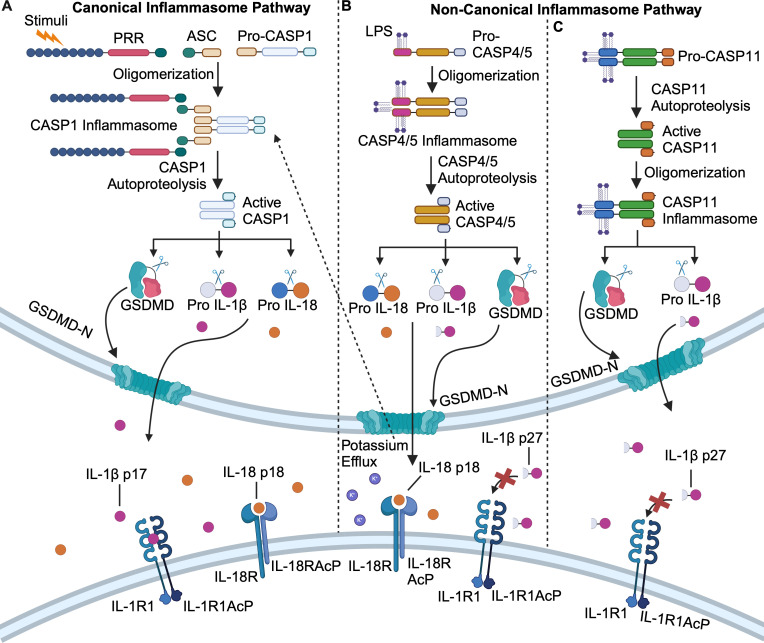
Overview of canonical and non-canonical inflammasome activation pathways. (**A**) Diverse stimuli are sensed by pattern-recognition receptors (PRRs) which recruit ASC and pro-caspase-1 (pro-CASP1) to form the canonical CASP1 inflammasome. CASP1 undergoes autoproteolytic maturation then cleaves and activates gasdermin D (GSDMD) and the interleukin family of cytokines IL-1β and IL-18. CASP1-mediated cleavage of IL-1β and IL-18 generates 17 and 18 kDa species, respectively, that are biologically active and signal through the IL-1 and IL18 receptors respectively. (**B**) Pro-caspases-4/5 (Pro-CASP4/5) bind to lipopolysaccharide (LPS) directly, leading to formation of the CASP4/5 non-canonical inflammasome. CASP4/5 undergo autoproteolytic maturation to cleave and activate GSDMD and IL-18 but cleave IL-1β into a biologically inactive 27 kDa species that does not signal to the IL-1 receptor. (**C**) Pro-caspase-11 (Pro-CASP11) binds directly to LPS and is first activated by autoproteolysis, and subsequently forms the non-canonical CASP11 inflammasome. Active CASP11 cleaves and activates GSDMD but cleaves and inactivates IL-1β by generating IL-1β p27. Image created using Biorender.

All caspases must be proteolytically activated to cleave their substrates. The induced-proximity model of caspase activation postulates that caspases are first autoproteolytically activated on multiprotein signaling complexes, allowing them to then process other substrates, including other caspases (15, 16). Activation of CASP1 occurs via autoproteolysis on the inflammasome, a signaling complex that assembles in response to diverse stimuli to initiate an inflammatory response (Fig. 1A) (17–19). Briefly, germline-encoded pattern recognition receptors (PRRs) detect stimuli known as pathogen-associated molecular patterns (PAMPs) or damage-associated molecular patterns (DAMPs) (18–21). When stimuli are detected, the cognate PRRs are activated and in some instances, recruit an adaptor protein known as ASC (apoptosis-associated speck-like protein containing a CARD) that recruits the pro-caspase-1 zymogen, forming the inflammasome (19–21). The increase in local concentration of caspase-1 is thought to trigger its autoproteolysis and activation, which subsequently cleaves the interleukin family of cytokines (IL-1β and IL-18) and the pore-forming GSDMD protein to mediate pyroptotic cell death (Fig. 1A) (22–25).

### Non-canonical inflammasomes

In contrast to the diverse array of stimuli detected by the PRRs in the canonical inflammasome pathway, non-canonical inflammasome activation is orchestrated by direct detection of intracellular lipopolysaccharide (LPS) from Gram-negative bacteria by CASP4/5 in humans (Fig. 1B) and CASP11 in mice (Fig. 1C) (12, 13, 19, 26–28). Upon binding LPS directly, CASP4/5/11 are thought to oligomerize and form inflammasomes, leading to autoproteolytic activation and subsequent cleavage of GSDMD to induce pyroptosis (Fig. 1B and C) (12, 13, 29, 30). A recent study visualized the CASP11 inflammasome by microscopy and demonstrated that CASP11 activity and autoproteolysis were both required for inflammasome formation (31). This study forces reconsideration of the current induced-proximity paradigm that the formation of the signaling complex induces caspase autoproteolysis and activation as CASP11 activation precedes inflammasome formation (Fig. 1C) (31). Notably, activation of the non-canonical pathway generally leads to activation of the canonical inflammasome pathway that activates CASP1 via detection of potassium efflux by the NLRP3 (NLR pyrin domain-containing protein 3) PRR (32). Thus, it has remained unclear whether CASP4/5/11 can directly contribute to inflammation by cleaving the interleukin family of cytokines. We and others recently demonstrated that human CASP4/5 can directly cleave IL-1β and IL-18, and mouse CASP11 can directly process IL-1β but not IL-18 (Fig. 1B and C) (33–35). However, non-canonical inflammasome-mediated processing of IL-18 activates the cytokine, but processing of IL-1β inactivates the cytokine by generating a 27 kDa species that does not signal through the IL-1 receptor (Fig. 1) (35, 36). It currently remains unclear if IL-1β p27 does not signal through the IL-1 receptor because it fails to bind, or if it binds but is unable to stimulate downstream signaling. Nevertheless, this study expands the verified substrates of CASP4/5/11 beyond GSDMD, suggesting other immune-modulating substrates of the non-canonical inflammasomes may exist.

### Peptide substrate libraries offer insights into caspase substrate specificities

**Fig 2:**
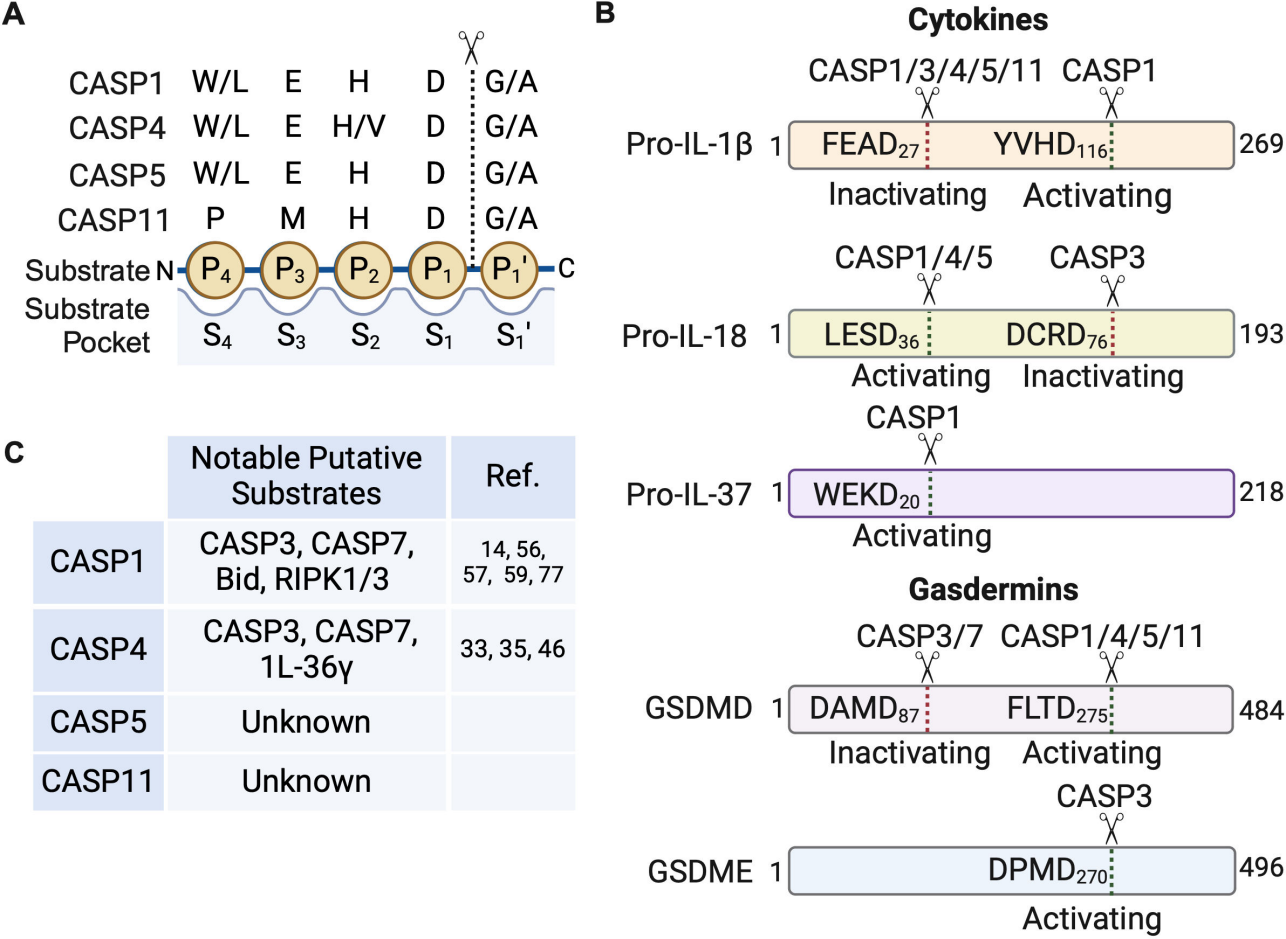
Schematic of inflammatory caspase peptide and protein substrate specificities. (**A**) The residue cleaved by caspases (dotted line) is designated P_1_ and the residues N-terminal to the cleaved peptide bond are numbered consecutively from the P_1_ position. Residues to the C-terminus of the peptide bond are also number consecutively and designate as prime (e.g., P_1_′) residues. These residues interact with the caspase substrate binding pockets, which have corresponding S designations (e.g., S_4_ – S_1_) to match the substrate convention, that help fine tune specificity. The preferred peptide substrate sequence and position of residues for the inflammatory caspases, as determined by positional scanning peptide library studies, are depicted. (**B**) Protein substrates indicating the amino acid sequence and position of caspase cleavage is shown. The biological consequence of cleavage at each specific site is also indicated. (**C**) Putative substrates that have potential crosstalk with other cell death pathways and the caspase reported to cleave those substrates are listed. These substrates need to be verified for direct processing in biologically relevant contexts. Image created using Biorender.

Much of the understanding of caspase substrate specificities stems from studies using synthetic substrate libraries (3, 4, 9, 37–43). Caspases cleave the scissile bond on the C-terminus of aspartic acid (Asp or D) residues, and this primary position is designated P_1_. The N-terminal residues preceding the processing site are designated P_4_ – P_2_ (Fig. 2A). Peptide libraries are typically generated by attaching a fluorescent reporter molecule to the C-terminus of the P_1_ Asp such that when cleaved, the reporter generates a florescent signal that can be detected. The P_1_ Asp is usually held constant while the remaining amino acid sequence of the tetrapeptide (P_4_ – P_2_) are substituted for all 20 amino acids. The amino acid composition of the tetrapeptide can profoundly influence cleavage specificity, but peptide substrates only inform on four residues and do not provide information about other residues that may contribute to recognition and processing.

In native protein substrates, the identity of the residues on the C-terminus of the P_1_ Asp can also influence substrate recognition. In place of the fluorescent reporter, the residues following the P_1_ Asp are generally designated P_1_′–P_4_′ (Fig. 2A). Notably, recent studies in which the tetrapeptide sequence was extended to include the P_1_′–P_4_′ position has offered new insights into the substrate preference of mouse CASP11 (37). As putative substrates interact with the caspase substrate binding pockets, designated S_4_–S_1_ (Fig. 2A), extending the peptide libraries beyond four amino acids offer more contacts that can influence specificity. Indeed, including the P_1_′–P_4_′ residues in a peptide substrate increased catalysis by CASP11, suggesting that CASP11 may need more contacts with putative substrates to facilitate processing (37).

The inflammatory caspases (CASP1/4/5) share similar substrate specificities, based on work with the positional scanning peptide libraries (Fig. 2A). Caspase-1 prefers hydrophobic and bulky residues (tryptophan/leucine or W/L) in the P_4_ position, which are well tolerated by a large S_4_ substrate binding pocket (40, 41). Glutamate (E) is preferred in the P_3_ position and histidine (H) in the P_2_ position. All caspases have a strong preference for aspartate (D) in the P_1_ position and small uncharged residues like glycine (G) or Alanine (A) in the P1′ position, making WEHD the optimal peptide substrate for CASP1 (Fig. 2B). Strikingly, CASP1 cleaved the WEHD tetrapeptide substrate at a rate that is ~12-fold faster than YVHD, the tetrapeptide sequence found in the native substrate, IL-1β, and ~22-fold faster than IL-1β itself (11, 41). Similar to CASP1, CASP4/5 also prefer (W/L)EHD (40) and LEVD has also been reported to be an optimal tetrapeptide sequence for CASP1 and CASP4 (38). Much of the earlier work excluded CASP11, but recent studies suggest that the optimal CASP11 sequence may be PMHD (42). Unnatural amino acids have also been incorporated and offer additional insights into caspase substrate specificities (43, 44). Using unnatural amino-acids, the authors were able to enhance processing by ~27-fold over the preferred PMHD tetrapeptide sequence, suggesting that CASP11 does not recognize the natural amino acids well and may have few native substrates. The optimal peptide sequence for inflammatory caspases are summarized in Fig. 2A.

### Inflammatory caspases utilize both active sites and exosites to recognize substrates

Extensive efforts have been made to determine the native caspase substrates in cells using a variety of approaches (4–8, 45, 46). These studies reveal that the inflammatory caspases have less substrates than apoptotic caspases. In 2010, Agard et al. performed a proteomic screen following caspase activation and identified 82 putative substrates of CASP1, three putative substrates of CASP4 and no substrates of CASP5 (6). Based on this work, the preferred protein-derived P4–P1 tetrapeptide sequence for CASP1 was FESD. Although this is the dominant motif, it is important to point out that other sequences can be cleaved. For example, the probability of a substrate having an LVPD sequence was nearly as high as having an FESD sequence (6). Amongst the identified substrates was GSDMD, which was cleaved ~50- to 500-fold faster than other substrates by CASP1. However, the functional impact of GSDMD processing on cell death and inflammation was not explored further. Intriguingly, GSDMD was not identified as a substrate of CASP4 in this screen, likely reflecting the inherently low catalytic activity of CASP4 compared to CASP1.

Recently, Davies et al. developed an antibody that specifically recognizes inflammatory caspase substrates with degenerate sequences preceding the P1 Asp (46). Using this antibody, they stimulated EA.hy926 endothelial cells with LPS to activate the CASP4 non-canonical inflammasome. EA.hy926 cells lack NLRP3 and are unable to activate CASP1 downstream of CASP4 activation, permitting specific detection of CASP4 substrates. The authors identified 328 putative protein substrates of CASP4 with a preferred tetrapeptide sequence of L, Q/E, T/S, and D. GSDMD was one of the identified substrates. Interestingly, many RNA binding proteins that are involved in RNA splicing and regulation of gene silencing by RNA were identified, suggesting that the non-canonical inflammasomes may modulate RNA splicing, transcription, and translation during bacterial infections to promote pyroptosis. Future work is needed to validate these substrates as well as determine the factors that engender specificity for the inflammatory caspases and the functional consequences of their cleavage with respect to pathogen defense. To our knowledge, proteomic screens to identify protein substrates of CASP5 and CASP11 have not been reported and would offer further insight into the inflammatory caspase substrate specificities and functions.

The most well-known and best-characterized substrates of the inflammatory caspases are GSDMD, IL-1β, and IL-18 (Fig. 2B) (11–14, 21, 47, 48). Recently, we and others have contributed to revealing the molecular basis of GSDMD, IL-1β, and IL-18 recognition by inflammatory caspases (33–35, 49, 50). Human GSDMD is cleaved at D275 by CASP1/4/5/11 to yield the pore-forming N-terminal domain (Fig. 2B). Based on insights gained from the extensive work using peptide substrate libraries, it is thought that the tetrapeptide sequence is what determines substrate specificity, but GSDMD appears to be an exception to this. Structural studies demonstrated that GSDMD is recognized by inflammatory caspases via an exosite and the GSDMD tetrapeptide sequence has no impact on processing (49, 50). Exosites are substrate-binding regions that are distinct from the enzyme active site. These sites can be critical determinants of substrate specificity, sometimes contributing more than the active site, as is the case with GSDMD. Thus, exosites can supersede specificity determinants derived from synthetic peptides. Intriguingly, CASP4 employs the same exosite used to bind GSDMD to recognize IL-18 (33, 34). However, unlike GSDMD, CASP1/4/5 interact with IL-18 using two distinct interfaces. In addition to the exosite, a series of electrostatic interactions at the active site confer specificity for IL-18, permitting CASP1/4/5 processing at D36 to generate the active cytokine in a tetrapeptide sequence-dependent manner (Fig. 2B) (33–35). IL-18 harbors an LESD tetrapeptide sequence, consistent with the preferred tetrapeptide sequence reported for CASP4 (46). In contrast to CASP4/5, the mouse ortholog, CASP11, is unable to process mouse IL-18 due to steric clashes and a lack of the proper electrostatic interactions with IL-18 at the active site, indicating that there may be species-specific determinants of substrate specificity (33, 34). While CASP1 cleaves IL-1β at D116 to generate the active cytokine, both human and mouse non-canonical inflammasomes (CASP4/5/11) cleave IL-1β at D27 to generate an inactive fragment that deactivates IL-1β signaling (Fig. 2B) (35). We demonstrated that the YVHD tetrapeptide sequence adjacent to the D116 cleavage site confers specificity for IL-1β processing into the active cytokine. Changing the YVHD sequence in IL-1β to the LESD sequence found in IL-18, which is recognized and processed by CASP4/5, permits CASP4/5-mediated processing at D116 to generate active IL-1β (35). Although no structure exists of IL-1β in complex with inflammatory caspases, it is likely that the active site and exosite will be the determinants of substrate specificity, like IL-18. It is tempting to speculate that many of the inflammatory caspase substrates will utilize the two interfaces (active site and exosite) to facilitate substrate recognition and binding except for GSDMD, which needs to execute rapid cell lysis and may not benefit from a bimodal recognition mechanism.

For nearly three decades, it was believed that only CASP1 can directly process IL-1β and IL-18. The finding that activation of the non-canonical inflammasome pathway leads to direct processing of IL-18 and IL-1β redefines our understanding of inflammation induced by inflammatory caspases (33–35). As a result, a panel of cytokines were recently tested for processing by inflammatory caspases and this work suggests that perhaps IL-1β and IL-18 may be the only cytokine substrates of CASP4/5 (33, 35). Contrary to prior reports, IL-1α was not identified as a substrate of the inflammatory caspases, but rather, it is cleaved by calpains downstream of inflammasome activation (35, 51). Pro-IL-36γ was processed by CASP4 *in vitro,* albeit slowly compared to IL-1β and IL-18, and is yet to be demonstrated *in vivo*, making it unclear if it is a true substrate (33). Pro-IL-37 was previously reported to be processed at D20 when macrophages were stimulated with LPS, which was attributed to CASP1, as the bioactivity of IL-37 was abrogated in ASC and NLRP3-deficient cells (Fig. 2B) (52, 53). In agreement with pro-IL-37 being a CASP1 substrate, recent *in vitro* work failed to detect CASP4-mediated processing of pro-IL-37 (33). More work is needed to clearly define the cytokine substrate profile of inflammatory caspases as this has important ramifications for immune defense against invading microbes. The identification of caspase substrates is challenging as there are overlapping specificities and the caspases can activate other caspases. Furthermore, *in vitro* results may not always recapitulate in cell findings if other factors are needed to facilitate substrate recognition and processing, such as localization to a particular macromolecular signaling structure like the inflammasome or cellular compartment. Thus, more studies are needed to identify caspase substrates, understand what determines specificities, and the biological relevance of substrate processing.

### Crosstalk between inflammatory and apoptotic caspases

An emerging theme is that significant crosstalk exists between the inflammatory and apoptotic signaling cascades, which allows for cross-regulation between different cell death pathways. Apoptosis is an immunologically silent type of programmed cell death that is initiated through two mechanisms, categorized as intrinsic and extrinsic apoptotic pathways. Intrinsic apoptosis, or the mitochondrial pathway, involves the activation of caspase-9 (CASP9) and extrinsic apoptosis involves the activation of death receptors at the cell surface, leading to the activation of caspase-8 (CASP8). Active CASP8 or CASP9 then cleave and activate executioner caspases, CASP3, -6, and -7, which subsequently cleave what is estimated to be thousands of substrates to induce apoptotic cell death (3, 4, 8). However, understanding which substrates are critical executors and those that are bystander substrates remains a major challenge. Substrates of the executioner caspases include endonucleases that mediate DNA fragmentation, and proteases for membrane and cytoskeletal breakdown (54). The crosstalk between apoptotic and pyroptotic cell death is complex; inflammatory caspases and apoptotic caspases can both promote and inhibit the opposing cell death pathways. The subsequent sections will focus on the interplay between apoptotic and inflammatory caspases, shared substrates, and ongoing questions in the field.

### Inflammatory caspases activate apoptotic substrates

Over 20 years ago, Van den Craen et al. aimed to map the interactions between inflammatory and apoptotic caspases by systematically determining if murine caspases could undergo autoproteolysis when the active and pro-forms were incubated together *in vitro* and if the active caspases could cleave and activate the pro-form of other caspase zymogens (55). A nearly unidirectional cross-talk was observed in which inflammatory caspases could cleave and activate apoptotic caspases, but apoptotic caspases could not cleave inflammatory caspases, except for CASP8. Specifically, murine CASP1 and -11 could cleave CASP2, -3, -6, -7, and -8. Only CASP8 was able to cleave CASP1 and -11, as well as CASP2, -3, -6, -7, and -12, suggesting that CASP8 has a broad substrate profile and may regulate multiple cell death pathways (55).

A proteomic screen aimed at identifying CASP1 substrates identified CASP7 as 1 of the 20 putative novel substrates of CASP1, further suggesting that the inflammatory caspases can induce apoptosis (Fig. 2C) (56). Indeed, they confirmed that CASP1 processed and activated CASP7 at the canonical activation site, D198, when activated by inflammatory stimuli such as LPS + ATP and flagellated *Salmonella* infection in mouse macrophages (56). Interestingly, CASP3 activation, which typically occurs when CASP7 is activated during apoptosis, was not dependent on CASP1. In contrast, recent work suggests that CASP1 activates CASP3 and CASP7 in the absence of the pyroptotic substrate GSDMD (14, 57). He et al. demonstrated that in GSDMD-deficient RAW 264.7 mouse macrophages, activation of CASP1 led to an increase in CASP3, -7, and -8 activity, suggesting that the apoptotic caspases could be substrates of CASP1 and that apoptosis may serve as a backup cell death pathway if GSDMD-mediated pyroptosis is inactivated by pathogens (14). For example, Enterovirus 71 is known to inhibit pyroptosis by cleaving and inactivating the pore-forming function of GSDMD (58).

Consistent with the above observations in mouse macrophages, Taabazuing et al. reported that in the absence of GSDMD, CASP1 activation induced CASP3 and CASP7 activation in human THP1 monocytes and in RAW 264.7 macrophages (57). Also, in support of the ability of CASP1 to induce apoptosis, it was reported that CASP1 cleaves Bid (Fig. 2C), a key mediator of CASP8/9 activation, leading to CASP3/7 activation and apoptosis that progresses rapidly to secondary necrosis (59). Early research into the crosstalk between apoptotic and inflammatory caspases demonstrated a unidirectional relationship, whereby inflammatory CASP1 can cleave apoptotic caspases *in vitro*. However, given the lack of ability of apoptotic caspases to cleave inflammatory caspases, it remained unclear if apoptotic caspases could regulate inflammatory cell death. Strikingly, the work by Taabazuing et al. also demonstrated that active CASP3 and to a lesser extent, CASP7, cleave GSDMD at D87, a distinct cleavage site from the pore-forming D275 site cleaved by inflammatory caspases that inactivate GSDMD pore-forming function (Fig. 2B). Taken together, these results imply that while pyroptotic cell death also activates apoptotic cell death as a potential failsafe to inflammatory cell death, apoptotic cell death acts as a negative regulator of inflammatory cell death by deactivating the critical GSDMD substrate (57). Perhaps this ensures that cell death occurs in response to microbial stimuli but ensures that no inflammation is induced during homeostatic cell death, which typically proceeds through apoptosis. Consistent with the notion that the apoptotic pathway actively prevents inflammation, it was previously reported that CASP3 cleaves IL-18 at D71 (DMTD_71_) and D76 (DCRD_76_), and IL-1β at D27 (FEAD_27_) to generate biologically inactive cytokines (Fig. 2B) (36, 37, 60, 61). Interestingly, the human non-canonical inflammasomes (CASP4/5) were also linked to the activation of the apoptotic pathway (35, 46). A proteomic screen identified and validated CASP7 as a substrate of CASP4 in cells (46), and we recently demonstrated that CASP3 is activated in a CASP4/5-dependent manner in response to LPS transfection and *Salmonella* infection (Fig. 2C) (35). However, whether this is a direct processing event and if it occurs *in vivo,* remains to be determined.

### Apoptotic caspases can elicit gasdermin-mediated cell death

Paradoxically, while CASP3 cleaves GSDMD at D87 to inactivate GSDMD-mediated pyroptosis, CASP3 cleaves GSDME at D270 to liberate its N-terminus to form pores and mediate pyroptosis (Fig. 2B) (62). This raises an important question in the field: why does CASP3 inactivate GSDMD, but activate GSDME? Recent work suggests that the expression of GSDME can convert apoptosis to pyroptosis, implying that GSDME is likely cleaved by CASP3 faster than the apoptotic substrates (63, 64). Analysis of expression data for each gasdermin may provide critical insights into cell death mechanisms in different tissues and cell types. Intriguingly, the CASP3/GSDMD/GSDME axis is also conserved in other mammals and birds. In birds, GSDMA is cleaved by bird CASP1 (65). Like in humans, CASP3 also activates GSDME in birds and deactivates GSDMA (65). This study demonstrates the evolutionary conservation of the CASP1/CASP3 crosstalk whereby CASP1 activates a gasdermin (GSDMD in humans and GSDMA in birds), that is deactivated by CASP3. As activation of CASP1 results in cytokine activation and inflammation, perhaps CASP3-mediated inactivation of GSDMD, IL-1β, and IL-18 prevents excessive inflammation should the canonical pathway become activated as a result of potassium efflux through GSDME pores (37, 57, 60, 61, 66). The evolutionary conservation of these axes hints at an important biological preservation that suggests apoptotic cell death is intended to be immunologically silent since the cells die but actively inactivate the pyroptotic (immunostimulatory) signaling pathway. Consistent with this idea, it has recently been reported that gasdermin B (GSDMB) binds to CASP4 to enhance its activity, however, during apoptosis, activated CASP7 cleaves GSDMB at D91 to prevent GSDMB-mediated enhancement of non-canonical inflammasome pyroptosis (67, 68). The biological implications and kinetics of the different caspases for their respective substrates during crosstalk warrant further exploration.

### Caspase-8 has broad and complex substrate specificities

Caspase-8 is an initiator caspase that triggers apoptosis, but it also has a known role in regulating another type of lytic cell death known as necroptosis, and more recently, it has been shown to also induce gasdermin-mediated cell death (69–71). Earlier *in vitro* work demonstrated that CASP8 was the only apoptotic caspase that can cleave the inflammatory caspases (mouse CASP1/11), suggesting that CASP8 may share overlapping substrates and/or that the inflammatory caspases are substrates of CASP8 (72). Indeed, recent work by the Brodsky lab indicates that CASP8 can cleave and activate CASP1, and that activated CASP8 can cleave the inflammatory substrates GSDMD, IL-1β, and IL-18 during *Yersinia pestis* infection in mouse bone marrow-derived macrophages (BMDMs) (73–75). However, as CASP8 activates CASP1, the precise contributions of CASP1 and CASP8 on inflammatory substrate processing warrants further investigation. Interestingly, CASP8 was also reported to cleave gasdermin C (GSDMC) to induce pyroptosis, suggesting that under certain conditions, CASP8 may serve as a backup or complementary pathway to the inflammatory caspases (70, 71).

Consistent with the notion that caspase-8 may serve as a backup pathway to the inflammatory caspases, it was reported that activation of the NLRC4 (NLR family CARD-containing protein 4) and NLRP1b (NLR family pyrin domain-containing protein 1b) PRRs leads to recruitment of the ASC adaptor and CASP8 to induce apoptosis in CASP1-deficient BMDMs (76). Also in agreement, another study suggested that catalytically inactive CASP1 can serve as a scaffold to recruit ASC and CASP8 to induce apoptosis (25). Likewise, catalytically inactive CASP8 can also serve as a scaffold to recruit ASC and CASP1, leading to CASP1-dependent processing of GSDMD, CASP3, and CASP7 (77). CASP8 typically cleaves RIPK1 to inhibit necroptosis, and CASP8 inhibition is embryonically lethal (77, 78). Newton et al. observed CASP1-dependent processing of RIPK1 and RIPK3 (Fig. 2C) in CASP8 catalytically inactive (CASP8^C362A^) expressing embryos, supporting the idea that CASP8 has overlapping substrate specificities with the inflammatory caspases and implying there may be crosstalk between the pyroptotic, necroptotic, and apoptotic signaling pathways (77). However, whether CASP1 directly processes RIPK1 and RIPK3 remains unclear. Furthermore, although RIPK1 and RIPK3 processing in the CASP8^C362A^ expressing embryos prevents necroptosis, these mice still die in the perinatal stage due to CASP1 and CASP11-mediated pyroptosis, which results in severe inflammation. Specifically, the authors noted elevated levels of IL-1β and IL-18 in the CASP8^C362A^ embryos but why and how CASP1/11 are activated during development remains an open question (77). Collectively, these findings indicate that there is significant crosstalk between the apoptotic, necroptotic, and pyroptotic cell death pathways with CASP8 at the nexus, perhaps serving as a backup mechanism for CASP1 and vice versa.

### Conclusions and open questions

Since the discovery of GSDMD, tremendous progress has been made in understanding inflammatory caspase activation mechanisms and biological functions. However, our understanding of the inflammatory caspase substrate repertoire and what determines specificity has been limited. Encouragingly, recent work has revealed new insights into inflammatory caspase substrate recognition. Inflammatory caspases recognize the tetrapeptide sequence of their substrates at the active site (except for GSDMD) and utilize a conserved exosite to bind substrates, which facilitates processing. Both exosite-mediated interactions and active site specificity are needed to recognize and process substrates, and therefore protein substrate sequences are not always in agreement with the predicted specificities derived from peptide library screens. Thus, new approaches are needed to identify inflammatory caspase substrates, and in particular, substrates of CASP5 and CASP11, for which little is known. CASP11 has a very limited known substrate repertoire and because CASP5 appeared in evolution after CASP1/4, it may have evolved to serve a unique function that is distinct from CASP1/4 (36, 42). Given that IL-18 and IL-1β have only recently been identified as direct substrates of the non-canonical inflammasome pathway, the outlook is promising that we may identify new substrates of the non-canonical inflammasomes that are critical regulators of innate immunity. As substrate processing can lead to gain of function or loss of function, major question that needs to be addressed are: (i) what are the functional consequences of substrate cleavage events? (ii) which substrates are the critical drivers of biological outcomes and which are bystanders? and (iii) can substrates that are identified *in vitro* and in proteomic screens be validated *in vivo*? Overcoming the hurdle of overlapping substrate specificities and the low abundance of certain substrates that make them challenging to identify will provide key insights into caspase biology. Future proteomic screens in cells where all other caspases have been genetically ablated might prove useful in assigning substrate specificity and function.

## References

[1] Alnemri ES, Livingston DJ, Nicholson DW, Salvesen G, Thornberry NA, Wong WW, Yuan J. 1996. Human ICE/CED-3 protease nomenclature. Cell 87:171–171. doi:10.1016/s0092-8674(00)81334-3 8861900

[2] Fuentes-Prior P, Salvesen GS. 2004. The protein structures that shape caspase activity, specificity, activation and inhibition. Biochem J 384:201–232. doi:10.1042/BJ20041142 15450003 PMC1134104

[3] Green DR. 2022. Caspases and their substrates. Cold Spring Harb Perspect Biol 14:a041012. doi:10.1101/cshperspect.a041012 35232877 PMC8886984

[4] Julien O, Wells JA. 2017. Caspases and their substrates. Cell Death Differ 24:1380–1389. doi:10.1038/cdd.2017.44 28498362 PMC5520456

[5] Seaman JE, Julien O, Lee PS, Rettenmaier TJ, Thomsen ND, Wells JA. 2016. Cacidases: caspases can cleave after aspartate, glutamate and phosphoserine residues. Cell Death Differ 23:1717–1726. doi:10.1038/cdd.2016.62 27367566 PMC5041198

[6] Agard NJ, Maltby D, Wells JA. 2010. Inflammatory stimuli regulate caspase substrate profiles. Mol Cell Proteomics 9:880–893. doi:10.1074/mcp.M900528-MCP200 20173201 PMC2871421

[7] Araya LE, Soni IV, Hardy JA, Julien O. 2021. Deorphanizing caspase-3 and caspase-9 substrates in and out of apoptosis with deep substrate profiling. ACS Chem Biol 16:2280–2296. doi:10.1021/acschembio.1c00456 34553588 PMC9116730

[8] Crawford ED, Seaman JE, Agard N, Hsu GW, Julien O, Mahrus S, Nguyen H, Shimbo K, Yoshihara HAI, Zhuang M, Chalkley RJ, Wells JA. 2013. The degrabase: a database of proteolysis in healthy and apoptotic human cells. Mol Cell Prot 12:813–824. doi:10.1074/mcp.O112.024372 PMC3591672 23264352

[9] Stennicke HR, Renatus M, Meldal M, Salvesen GS. 2000. Internally quenched fluorescent peptide substrates disclose the subsite preferences of human caspases 1, 3, 6, 7 and 8. Biochem J 350 Pt 2:563–568. 10947972 PMC1221285

[10] Cerretti DP, Kozlosky CJ, Mosley B, Nelson N, Van Ness K, Greenstreet TA, March CJ, Kronheim SR, Druck T, Cannizzaro LA, Huebner K, Black RA. 1992. Molecular cloning of the interleukin- 1β converting enzyme. Science 256:97–100. doi:10.1126/science.1373520 1373520

[11] Thornberry NA, Bull HG, Calaycay JR, Chapman KT, Howard AD, Kostura MJ, Miller DK, Molineaux SM, Weidner JR, Aunins J, et al.. 1992. A novel heterodimeric cysteine protease is required for interleukin-1β processing in monocytes. Nature 356:768–774. doi:10.1038/356768a0 1574116

[12] Kayagaki N, Stowe IB, Lee BL, O’Rourke K, Anderson K, Warming S, Cuellar T, Haley B, Roose-Girma M, Phung QT, Liu PS, Lill JR, Li H, Wu J, Kummerfeld S, Zhang J, Lee WP, Snipas SJ, Salvesen GS, Morris LX, Fitzgerald L, Zhang Y, Bertram EM, Goodnow CC, Dixit VM. 2015. Caspase-11 cleaves gasdermin D for non-canonical Inflammasome signalling. Nature 526:666–671. doi:10.1038/nature15541 26375259

[13] Shi J, Zhao Y, Wang K, Shi X, Wang Y, Huang H, Zhuang Y, Cai T, Wang F, Shao F. 2015. Cleavage of GSDMD by inflammatory caspases determines pyroptotic cell death. Nature 526:660–665. doi:10.1038/nature15514 26375003

[14] He W, Wan H, Hu L, Chen P, Wang X, Huang Z, Yang Z-H, Zhong C-Q, Han J. 2015. Gasdermin D is an executor of pyroptosis and required for interleukin-1β secretion. Cell Res. 25:1285–1298. doi:10.1038/cr.2015.139 26611636 PMC4670995

[15] Bao Q, Shi Y. 2007. Apoptosome: a platform for the activation of initiator caspases. Cell Death Differ 14:56–65. doi:10.1038/sj.cdd.4402028 16977332

[16] Salvesen GS, Dixit VM. 1999. Caspase activation: the induced-proximity model. Proc Natl Acad Sci U S A 96:10964–10967. doi:10.1073/pnas.96.20.10964 10500109 PMC34227

[17] Martinon F, Burns K, Tschopp J. 2002. The inflammasome: a molecular platform triggering activation of inflammatory caspases and processing of proIL-beta. Mol Cell 10:417–426. doi:10.1016/s1097-2765(02)00599-3 12191486

[18] Taabazuing CY, Griswold AR, Bachovchin DA. 2020. The NLRP1 and CARD8 inflammasomes. Immunol Rev 297:13–25. doi:10.1111/imr.12884 32558991 PMC7483925

[19] Broz P, Dixit VM. 2016. Inflammasomes: mechanism of assembly, regulation and signalling. Nat Rev Immunol 16:407–420. doi:10.1038/nri.2016.58 27291964

[20] Lamkanfi M, Dixit VM. 2014. Mechanisms and functions of inflammasomes. Cell 157:1013–1022. doi:10.1016/j.cell.2014.04.007 24855941

[21] Barnett KC, Li S, Liang K, Ting JPY. 2023. A 360° view of the inflammasome: mechanisms of activation, cell death, and diseases. Cell 186:2288–2312. doi:10.1016/j.cell.2023.04.025 37236155 PMC10228754

[22] Boucher D, Monteleone M, Coll RC, Chen KW, Ross CM, Teo JL, Gomez GA, Holley CL, Bierschenk D, Stacey KJ, Yap AS, Bezbradica JS, Schroder K. 2018. Caspase-1 self-cleavage is an intrinsic mechanism to terminate inflammasome activity. J Exp Med 215:827–840. doi:10.1084/jem.20172222 29432122 PMC5839769

[23] Guey B, Bodnar M, Manié SN, Tardivel A, Petrilli V. 2014. Caspase-1 autoproteolysis is differentially required for NLRP1b and NLRP3 inflammasome function. Proc Natl Acad Sci USA 111:17254–17259. doi:10.1073/pnas.1415756111 25404286 PMC4260594

[24] Broz P, von Moltke J, Jones JW, Vance RE, Monack DM. 2010. Differential requirement for caspase-1 autoproteolysis in pathogen-induced cell death and cytokine processing. Cell Host Microbe 8:471–483. doi:10.1016/j.chom.2010.11.007 21147462 PMC3016200

[25] Ball DP, Taabazuing CY, Griswold AR, Orth EL, Rao SD, Kotliar IB, Vostal LE, Johnson DC, Bachovchin DA. 2020. Caspase-1 interdomain linker cleavage is required for pyroptosis. Life Sci Alliance 3:e202000664. doi:10.26508/lsa.202000664 32051255 PMC7025033

[26] Shi J, Zhao Y, Wang Y, Gao W, Ding J, Li P, Hu L, Shao F. 2014. Inflammatory caspases are innate immune receptors for intracellular LPS. Nature 514:187–192. doi:10.1038/nature13683 25119034

[27] Hagar JA, Powell DA, Aachoui Y, Ernst RK, Miao EA. 2013. Cytoplasmic LPS activates caspase-11: implications in TLR4-independent endotoxic shock. Science 341:1250–1253. doi:10.1126/science.1240988 24031018 PMC3931427

[28] Viganò E, Diamond CE, Spreafico R, Balachander A, Sobota RM, Mortellaro A. 2015. Human caspase-4 and caspase-5 regulate the one-step non-canonical inflammasome activation in monocytes. Nat Commun 6:8761. doi:10.1038/ncomms9761 26508369 PMC4640152

[29] Lee BL, Stowe IB, Gupta A, Kornfeld OS, Roose-Girma M, Anderson K, Warming S, Zhang J, Lee WP, Kayagaki N. 2018. Caspase-11 auto-proteolysis is crucial for noncanonical inflammasome activation. J Exp Med 215:2279–2288. doi:10.1084/jem.20180589 30135078 PMC6122968

[30] Ross C, Chan AH, Von Pein J, Boucher D, Schroder K. 2018. Dimerization and auto-processing induce caspase-11 protease activation within the non-canonical inflammasome. Life Sci Alliance 1:e201800237. doi:10.26508/lsa.201800237 30564782 PMC6284101

[31] Akuma DC, Wodzanowski KA, Schwartz Wertman R, Exconde PM, Vázquez Marrero VR, Odunze CE, Grubaugh D, Shin S, Taabazuing C, Brodsky IE. 2024. Catalytic activity and autoprocessing of murine caspase-11 mediate noncanonical inflammasome assembly in response to cytosolic LPS. Elife 13:e83725. doi:10.7554/eLife.83725 38231198 PMC10794067

[32] Kayagaki N, Warming S, Lamkanfi M, Vande Walle L, Louie S, Dong J, Newton K, Qu Y, Liu J, Heldens S, Zhang J, Lee WP, Roose-Girma M, Dixit VM. 2011. Non-canonical inflammasome activation targets caspase-11. Nature 479:117–121. doi:10.1038/nature10558 22002608

[33] Devant P, Dong Y, Mintseris J, Ma W, Gygi SP, Wu H, Kagan JC. 2023. Structural insights into cytokine cleavage by inflammatory caspase-4. Nature 624:451–459. doi:10.1038/s41586-023-06751-9 37993712 PMC10807405

[34] Shi X, Sun Q, Hou Y, Zeng H, Cao Y, Dong M, Ding J, Shao F. 2023. Recognition and maturation of IL-18 by caspase-4 noncanonical inflammasome. Nature 624:442–450. doi:10.1038/s41586-023-06742-w 37993714

[35] Exconde PM, Hernandez-Chavez C, Bourne CM, Richards RM, Bray MB, Lopez JL, Srivastava T, Egan MS, Zhang J, Yoo W, Shin S, Discher BM, Taabazuing CY. 2023. The tetrapeptide sequence of IL-18 and IL-1β regulates their recruitment and activation by inflammatory caspases. Cell Rep 42:113581. doi:10.1016/j.celrep.2023.113581 38103201 PMC11158830

[36] Bibo-Verdugo B, Joglekar I, Karadi Giridhar MN, Ramirez ML, Snipas SJ, Clark AC, Poreba M, Salvesen GS. 2022. Resurrection of an ancient inflammatory locus reveals switch to caspase-1 specificity on a caspase-4 scaffold. J Biol Chem 298:101931. doi:10.1016/j.jbc.2022.101931 35427646 PMC9144055

[37] Bibo-Verdugo B, Snipas SJ, Kolt S, Poreba M, Salvesen GS. 2020. Extended subsite profiling of the pyroptosis effector protein gasdermin D reveals a region recognized by inflammatory caspase-11. J Biol Chem 295:11292–11302. doi:10.1074/jbc.RA120.014259 32554464 PMC7415983

[38] Talanian RV, Quinlan C, Trautz S, Hackett MC, Mankovich JA, Banach D, Ghayur T, Brady KD, Wong WW. 1997. Substrate specificities of caspase family proteases. J Biol Chem 272:9677–9682. doi:10.1074/jbc.272.15.9677 9092497

[39] Poreba M, Strózyk A, Salvesen GS, Drag M. 2013. Caspase substrates and inhibitors. Cold Spring Harb Perspect Biol 5:a008680. doi:10.1101/cshperspect.a008680 23788633 PMC3721276

[40] Thornberry NA, Rano TA, Peterson EP, Rasper DM, Timkey T, Garcia-Calvo M, Houtzager VM, Nordstrom PA, Roy S, Vaillancourt JP, Chapman KT, Nicholson DW. 1997. A combinatorial approach defines specificities of members of the caspase family and granzyme B. functional relationships established for key mediators of apoptosis. J Biol Chem 272:17907–17911. doi:10.1074/jbc.272.29.17907 9218414

[41] Rano TA, Timkey T, Peterson EP, Rotonda J, Nicholson DW, Becker JW, Chapman KT, Thornberry NA. 1997. A combinatorial approach for determining protease specificities: application to interleukin-1β converting enzyme (ICE). Chem Biol 4:149–155. doi:10.1016/S1074-5521(97)90258-1 9190289

[42] Ramirez MLG, Poreba M, Snipas SJ, Groborz K, Drag M, Salvesen GS. 2018. Extensive peptide and natural protein substrate screens reveal that mouse caspase-11 has much narrower substrate specificity than caspase-1. J Biol Chem 293:7058–7067. doi:10.1074/jbc.RA117.001329 29414788 PMC5936834

[43] Poreba M, Salvesen GS, Drag M. 2017. Synthesis of a hycosul peptide substrate library to dissect protease substrate specificity. Nat Protoc 12:2189–2214. doi:10.1038/nprot.2017.091 28933778

[44] Poreba M, Kasperkiewicz P, Snipas SJ, Fasci D, Salvesen GS, Drag M. 2014. Unnatural amino acids increase sensitivity and provide for the design of highly selective caspase substrates. Cell Death Differ 21:1482–1492. doi:10.1038/cdd.2014.64 24832467 PMC4131180

[45] Agard NJ, Mahrus S, Trinidad JC, Lynn A, Burlingame AL, Wells JA. 2012. Global kinetic analysis of proteolysis via quantitative targeted proteomics. Proc Natl Acad Sci USA 109:1913–1918. doi:10.1073/pnas.1117158109 22308409 PMC3277568

[46] Davies CW, Stowe I, Phung QT, Ho H, Bakalarski CE, Gupta A, Zhang Y, Lill JR, Payandeh J, Kayagaki N, Koerber JT. 2021. Discovery of a caspase cleavage motif antibody reveals insights into noncanonical inflammasome function. Proc Natl Acad Sci USA 118:1. doi:10.1073/pnas.2018024118 PMC8000503 33723046

[47] Puren AJ, Fantuzzi G, Dinarello CA. 1999. Gene expression, synthesis, and secretion of interleukin 18 and interleukin 1β are differentially regulated in human blood mononuclear cells and mouse spleen cells. Proc Natl Acad Sci U S A 96:2256–2261. doi:10.1073/pnas.96.5.2256 10051628 PMC26770

[48] Fantuzzi G, Puren AJ, Harding MW, Livingston DJ, Dinarello CA. 1998. Interleukin-18 regulation of interferon γ production and cell proliferation as shown in interleukin-1β–converting enzyme (caspase-1)-deficient mice. Blood 91:2118–2125. 9490698

[49] Liu Z, Wang C, Yang J, Chen Y, Zhou B, Abbott DW, Xiao TS. 2020. Caspase-1 engages full-length gasdermin D through two distinct interfaces that mediate caspase recruitment and substrate cleavage. Immunity 53:106–114. doi:10.1016/j.immuni.2020.06.007 32553275 PMC7382298

[50] Wang K, Sun Q, Zhong X, Zeng M, Zeng H, Shi X, Li Z, Wang Y, Zhao Q, Shao F, Ding J. 2020. Structural mechanism for GSDMD targeting by autoprocessed caspases in pyroptosis. Cell 180:941–955. doi:10.1016/j.cell.2020.02.002 32109412

[51] Tsuchiya K, Hosojima S, Hara H, Kushiyama H, Mahib MR, Kinoshita T, Suda T. 2021. Gasdermin D mediates the maturation and release of IL-1α downstream of inflammasomes. Cell Rep 34:108887. doi:10.1016/j.celrep.2021.108887 33761363

[52] Bulau A-M, Nold MF, Li S, Nold-Petry CA, Fink M, Mansell A, Schwerd T, Hong J, Rubartelli A, Dinarello CA, Bufler P. 2014. Role of caspase-1 in nuclear translocation of IL-37, release of the cytokine, and IL-37 inhibition of innate immune responses. Proc Natl Acad Sci U S A 111:2650–2655. doi:10.1073/pnas.1324140111 24481253 PMC3932872

[53] Chan AH, Schroder K. 2020. Inflammasome signaling and regulation of interleukin-1 family cytokines. J Exp Med 217:e20190314. doi:10.1084/jem.20190314 31611248 PMC7037238

[54] Elmore S. 2007. Apoptosis: a review of programmed cell death. Toxicol Pathol 35:495–516. doi:10.1080/01926230701320337 17562483 PMC2117903

[55] Van de Craen M, Declercq W, Van den brande I, Fiers W, Vandenabeele P. 1999. The proteolytic procaspase activation network: an in vitro analysis. Cell Death Differ 6:1117–1124. doi:10.1038/sj.cdd.4400589 10578181

[56] Lamkanfi M, Kanneganti T-D, Van Damme P, Vanden Berghe T, Vanoverberghe I, Vandekerckhove J, Vandenabeele P, Gevaert K, Núñez G. 2008. Targeted peptidecentric proteomics reveals caspase-7 as a substrate of the caspase-1 inflammasomes. Mol Cell Proteomics 7:2350–2363. doi:10.1074/mcp.M800132-MCP200 18667412 PMC2596343

[57] Taabazuing CY, Okondo MC, Bachovchin DA. 2017. Pyroptosis and apoptosis pathways engage in bidirectional crosstalk in monocytes and macrophages. Cell Chem Biol 24:507–514. doi:10.1016/j.chembiol.2017.03.009 28392147 PMC5467448

[58] Lei X, Zhang Z, Xiao X, Qi J, He B, Wang J. 2017. Enterovirus 71 inhibits pyroptosis through cleavage of gasdermin D. J Virol 91:e01069-17. doi:10.1128/JVI.01069-17 28679757 PMC5571240

[59] Heilig R, Dilucca M, Boucher D, Chen KW, Hancz D, Demarco B, Shkarina K, Broz P. 2020. Caspase-1 cleaves bid to release mitochondrial SMAC and drive secondary necrosis in the absence of GSDMD. Life Sci Alliance 3:e202000735. doi:10.26508/lsa.202000735 32345661 PMC7190276

[60] Gu Y, Kuida K, Tsutsui H, Ku G, Hsiao K, Fleming MA, Hayashi N, Higashino K, Okamura H, Nakanishi K, Kurimoto M, Tanimoto T, Flavell RA, Sato V, Harding MW, Livingston DJ, Su MS. 1997. Activation of interferon-gamma inducing factor mediated by interleukin-1 beta converting enzyme. Science 275:206–209. doi:10.1126/science.275.5297.206 8999548

[61] Akita K, Ohtsuki T, Nukada Y, Tanimoto T, Namba M, Okura T, Takakura-Yamamoto R, Torigoe K, Gu Y, Su MS, Fujii M, Satoh-Itoh M, Yamamoto K, Kohno K, Ikeda M, Kurimoto M. 1997. Involvement of caspase-1 and caspase-3 in the production and processing of mature human interleukin 18 in monocytic THP.1 cells. J Biol Chem 272:26595–26603. doi:10.1074/jbc.272.42.26595 9334240

[62] Wang Y, Gao W, Shi X, Ding J, Liu W, He H, Wang K, Shao F. 2017. Chemotherapy drugs induce pyroptosis through caspase-3 cleavage of a gasdermin. Nature 547:99–103. doi:10.1038/nature22393 28459430

[63] Bourne CM, Taabazuing CY. 2024. Harnessing pyroptosis for cancer immunotherapy. Cells 13:346. doi:10.3390/cells13040346 38391959 PMC10886719

[64] Zhang Z, Zhang Y, Xia S, Kong Q, Li S, Liu X, Junqueira C, Meza-Sosa KF, Mok TMY, Ansara J, Sengupta S, Yao Y, Wu H, Lieberman J. 2020. Gasdermin E suppresses tumour growth by activating anti-tumour immunity. Nature 579:415–420. doi:10.1038/s41586-020-2071-9 32188940 PMC7123794

[65] Billman ZP, Kovacs SB, Wei B, Kang K, Cissé OH, Miao EA. 2023. Caspase-1 activates gasdermin A in non-mammals. Immunology. doi:10.1101/2023.09.28.559989 PMC10948149 38497531

[66] Rühl S, Broz P. 2015. Caspase-11 activates a canonical NLRP3 inflammasome by promoting K+ efflux. Eur J Immunol 45:2927–2936. doi:10.1002/eji.201545772 26173909

[67] Li X, Zhang T, Kang L, Xin R, Sun M, Chen Q, Pei J, Chen Q, Gao X, Lin Z. 2023. Apoptotic caspase-7 activation inhibits non-canonical pyroptosis by GSDMB cleavage. Cell Death Differ 30:2120–2134. doi:10.1038/s41418-023-01211-3 37591921 PMC10482963

[68] Chen Q, Shi P, Wang Y, Zou D, Wu X, Wang D, Hu Q, Zou Y, Huang Z, Ren J, Lin Z, Gao X. 2019. GSDMB promotes non-canonical pyroptosis by enhancing caspase-4 activity. J Mol Cell Biol 11:496–508. doi:10.1093/jmcb/mjy056 30321352 PMC6734491

[69] Fritsch M, Günther SD, Schwarzer R, Albert M-C, Schorn F, Werthenbach JP, Schiffmann LM, Stair N, Stocks H, Seeger JM, Lamkanfi M, Krönke M, Pasparakis M, Kashkar H. 2019. Caspase-8 is the molecular switch for apoptosis, necroptosis and pyroptosis. Nature 575:683–687. doi:10.1038/s41586-019-1770-6 31748744

[70] Zhang JY, Zhou B, Sun RY, Ai YL, Cheng K, Li FN, Wang BR, Liu FJ, Jiang ZH, Wang WJ, Zhou D, Chen HZ, Wu Q. 2021. The metabolite alpha-KG induces GSDMC-dependent pyroptosis through death receptor 6-activated caspase-8. Cell Res 31:980–997. doi:10.1038/s41422-021-00506-9 34012073 PMC8410789

[71] Hou J, Zhao R, Xia W, Chang CW, You Y, Hsu JM, Nie L, Chen Y, Wang YC, Liu C, Wang WJ, Wu Y, Ke B, Hsu JL, Huang K, Ye Z, Yang Y, Xia X, Li Y, Li CW, Shao B, Tainer JA, Hung MC. 2020. PD-L1-mediated gasdermin C expression switches apoptosis to pyroptosis in cancer cells and facilitates tumour necrosis. Nat Cell Biol 22:1264–1275. doi:10.1038/s41556-020-0575-z 32929201 PMC7653546

[72] Van de Craen M, Declercq W, Van den brande I, Fiers W, Vandenabeele P. 1999. The proteolytic procaspase activation network: an in vitro analysis. Cell Death Differ. 6:1117–1124. doi:10.1038/sj.cdd.4400589 10578181

[73] Demarco B, Grayczyk JP, Bjanes E, Le Roy D, Tonnus W, Assenmacher C-A, Radaelli E, Fettrelet T, Mack V, Linkermann A, Roger T, Brodsky IE, Chen KW, Broz P. 2020. Caspase-8-dependent gasdermin D cleavage promotes antimicrobial defense but confers susceptibility to TNF-induced lethality. Sci Adv 6:eabc3465. doi:10.1126/sciadv.abc3465 33208362 PMC7673803

[74] Wertman RS, Go CK, Saller BS, Groß O, Scott P, Brodsky IE. 2023. Sequentially activated death complexes regulate pyroptosis and IL-1β release in response to Yersinia blockade of immune signaling. bioRxiv:2023.09.14.557714. doi:10.1101/2023.09.14.557714

[75] Philip NH, Dillon CP, Snyder AG, Fitzgerald P, Wynosky-Dolfi MA, Zwack EE, Hu B, Fitzgerald L, Mauldin EA, Copenhaver AM, Shin S, Wei L, Parker M, Zhang J, Oberst A, Green DR, Brodsky IE. 2014. Caspase-8 mediates caspase-1 processing and innate immune defense in response to bacterial blockade of NF-κB and MAPK signaling. Proc Natl Acad Sci U S A 111:7385–7390. doi:10.1073/pnas.1403252111 24799700 PMC4034241

[76] Van Opdenbosch N, Van Gorp H, Verdonckt M, Saavedra PHV, de Vasconcelos NM, Gonçalves A, Vande Walle L, Demon D, Matusiak M, Van Hauwermeiren F, D’Hont J, Hochepied T, Krautwald S, Kanneganti T-D, Lamkanfi M. 2017. Caspase-1 engagement and TLR-induced c-FLIP expression suppress ASC/caspase-8-dependent apoptosis by inflammasome sensors NLRP1b and NLRC4. Cell Rep 21:3427–3444. doi:10.1016/j.celrep.2017.11.088 29262324 PMC5746600

[77] Newton K, Wickliffe KE, Maltzman A, Dugger DL, Reja R, Zhang Y, Roose-Girma M, Modrusan Z, Sagolla MS, Webster JD, Dixit VM. 2019. Activity of caspase-8 determines plasticity between cell death pathways. Nature 575:679–682. doi:10.1038/s41586-019-1752-8 31723262

[78] Newton K, Wickliffe KE, Dugger DL, Maltzman A, Roose-Girma M, Dohse M, Kőműves L, Webster JD, Dixit VM. 2019. Cleavage of RIPK1 by caspase-8 is crucial for limiting apoptosis and necroptosis. Nature 574:428–431. doi:10.1038/s41586-019-1548-x 31511692

